# EGFR T790M-Positive Lung Adenocarcinoma Metastases to the Pituitary Gland Causing Adrenal Insufficiency: A Case Report

**DOI:** 10.1155/2018/2349021

**Published:** 2018-05-31

**Authors:** Michael L. Adashek, Kenneth Miller, Arit A. Silpasuvan

**Affiliations:** ^1^Department of Internal Medicine, Sinai Hospital, Baltimore, MD, USA; ^2^University of Maryland Marlene & Stewart Greenebaum Comprehensive Cancer Center, Baltimore, MD, USA; ^3^Department of Endocrinology, Sinai Hospital, Baltimore, MD, USA

## Abstract

A 64-year-old man, with history of micropapillary thyroid cancer and epidermal growth factor receptor-positive lung adenocarcinoma with no evidence of active disease for 3 years after chemotherapy and radiation on erlotinib, presented with fatigue, nausea, lack of appetite, and xeroderma. A screening magnetic resonance image of the patient's head demonstrated a new bilateral pituitary mass. Initial evaluation revealed low morning cortisol, and the patient was diagnosed with adrenal insufficiency. His symptoms rapidly improved with maintenance glucocorticoids. Soon thereafter, the patient developed an acute visual deficit secondary to enlargement of the pituitary mass, and biopsy revealed EGFR T790M positive metastatic lung adenocarcinoma. Hence, we present a rare case of metastatic lung adenocarcinoma to the pituitary causing secondary adrenal insufficiency.

## 1. Introduction

Adrenal insufficiency (AI) is a broadly encompassing term for inadequate physiologic corticosteroid production. Primary AI is due to adrenal gland inadequacy, whereas secondary AI is due to disruption of the hypothalamus or pituitary in the hypothalamus-pituitary-adrenal (HPA) axis. We present a case of a man who presented with new fatigue, nausea, lack of appetite, and xeroderma who was found to have a new pituitary mass resulting in secondary AI.

## 2. Case Presentation

### 2.1. Presentation

**Figure 1:**
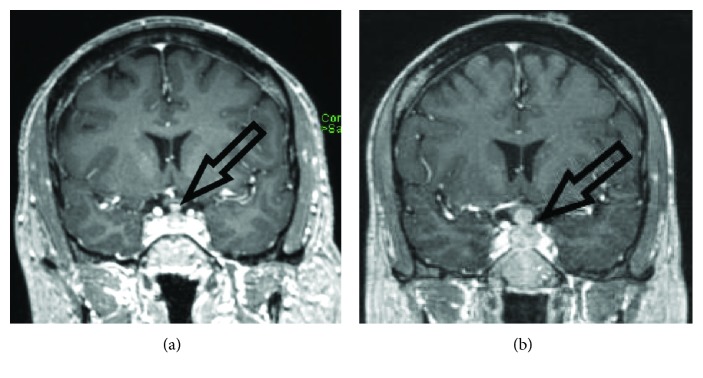
Brain magnetic resonance imaging demonstrating enlarging pituitary mass. (a) T1-weighted MRI demonstrating enlarged pituitary gland containing a hypovascular 10.5 × 7.5 mm mass lesion with thickening of the pituitary infundibulum without intracranial hemorrhage or extra-axial fluid collection. (b) Imaging one month later with T1-weighted MRI demonstrating a 22.0 × 12.0 mm bilobed mass with 8 mm suprasellar extension exerting mass effect on overlying optic chiasm.

A 64-year-old man with a medical history of micropapillary thyroid cancer and stage IIIb lung adenocarcinoma with no evidence of active disease for 3 years after chemotherapy and radiation presented with subjective complaints of new onset fatigue, nausea, scalp tenderness, and xeroderma. His medications included gabapentin 300 mg four times a day for chemotherapy-induced neuropathy, erlotinib 150 mg once daily for epidermal growth factor receptor- (EGFR-) positive lung adenocarcinoma, and omeprazole 40 mg once daily for subjective gastroesophageal reflux disease. A screening magnetic resonance image of the head revealed a new hypovascular pituitary mass measuring approximately 1 cm by 0.8 cm (Figure 1(a)).

### 2.2. Assessment

On examination, the patient's vital signs were within normal limits. On physical exam, xeroderma was appreciated in all extremities. Finger size was proportional and no prognathism, acromegaly, or Cushingoid features were appreciated. The cardiopulmonary exam was normal.

Initial lab values demonstrated normal free triiodothyronine (T3) of 2.4 pg/mL (normal range (NR) 1.8–4.2 pg/mL), normal T3 of 86 ng/dL (NR 70–172 ng/dL), and normal free thyroxine of 1.00 ng/dL (NR 0.84–1.68 ng/dL). Prolactin was elevated at 28.9 ng/mL (NR 2.5–17.0 ng/mL). The patient's morning cortisol was immeasurably low at <1.0 mcg/dL (NR > 10 mcg/dL) as was the patient's testosterone level at <20 ng/dL (280–1100 ng/dL). Luteinizing hormone was low at 0.05 mIU/mL (NR 1.8–12.0 mIU/L).

The patient was started on prednisone 20 mg by mouth daily, at which point he noticed immediate improvement in his energy and appetite as well as decrease in his nausea. For chronic steroid replacement therapy, the patient's treatment was changed from prednisone to hydrocortisone 20 mg of hydrocortisone in the morning and 10 mg in the evening. The patient was additionally instructed about the dangers of adrenal crisis and told to increase his hydrocortisone to 90 mg daily if acutely ill.

### 2.3. Diagnosis

Within a month of initial diagnoses, the patient suffered acute visual bilateral field cut and loss of peripheral vision. A repeat MRI demonstrated rapid enlargement of his pituitary mass, nearly doubled in size and described as a 2.2 cm by 1.2 cm mass impinging on the overlying optic chiasm (Figure 1(b)).

The patient subsequently underwent transsphenoidal resection of his pituitary mass. Gross histology characterized the mass as firm and fibrous. Macroscopic analysis revealed metastatic lung adenocarcinoma described as adenohypophysis fibrosis. Further histologic analysis revealed positive identification of cytokeratin 7, TTF-1, Ki-67, and epidermal growth factor receptor (EGFR) positive with EGFR gene nucleotide change demonstrating T790M and L858R positivity. This histopathology demonstrated further EGFR mutation of the patient's known history of lung adenocarcinoma which initially was only positive for EGFR mutation L585R.

After transsphenoidal resection and subsequent whole-brain radiation, further results demonstrated a continued low morning cortisol at <1.0 mcg/dL (NR > 10 mcg/dL) and testosterone level at <20 ng/dL (280–1100 ng/dL). Luteinizing hormone was low at <0.1 mIU/mL (NR 1.8–12.0 mIU/L) as was follicle-stimulating hormone 0.8 mIU/mL (NR 1.5–12.4 mIU/mL). Free T4 was low at 0.65 ng/dL (NR 0.84–1.68 ng/dL) and thyroid-stimulating hormone was low at 0.019 MCI/mL (NR 0.4–4.0 MCI/mL). Prolactin was lower than previous but still elevated at 14.9 ng/mL (NR 2.5–17.0 ng/mL).

## 3. Discussion

Annually, 200,000 new patients are diagnosed with brain metastasis (BM), making BM the most frequent cause of intercranial neoplasm in adults in the United States. An estimated 20–40% of adult patients with systemic malignancies will develop BM, and of those, a further 20% will become symptomatic over the course of their disease. Lung cancer comprises the majority of these BM cases (50%), followed by breast cancer (20–30%) and melanoma (5–10%) [1]. Complications of brain metastases are one of the chief causes of mortality and morbidity in patients with non-small cell lung cancer [2]. Adrenal insufficiency (AI) secondary to metastatic malignancy is highly unusual and has been reported in under 100 cases in English literature [3].

Adrenal insufficiency (AI) stems from inadequate physiologic steroid production and is most commonly a result of discontinuation of long-term glucocorticoid treatment. The signs of adrenal insufficiency are wide and varied including orthostatic hypotension, altered mental status, nausea and vomiting, abdominal pain, weight loss, and salt craving, many of which can be attributed to fluid losses from reduced mineralocorticoid function [4]. Physical signs of AI can include Cushingoid appearance: thinning skin, striae, obesity, muscle wasting, and psychiatric disturbance, all of which may indicate prior chronic iatrogenic steroid exposure. The treatment is physiologic replacement, with hydrocortisone demonstrating decreased LDL levels compared to prednisone making hydrocortisone the replacement therapy of choice [5]. The patient demonstrated initial rapid symptomatic improvement with physiologic corticosteroid replacement. Of note, AI develops only in patients with bilateral hypothalamic-pituitary-adrenal (HPA) axis involvement. However, HPA metastasis typically results in an excitation of the HPA system leading to increased cortisol levels, making our patient's presentation of AI secondary to pituitary metastases an unusual one [6].

Radiologically, it is difficult to differentiate BM from primary intercranial malignancies. BM is typically found in the cerebral hemispheres (80%), followed by the cerebellum (15%) and brainstem (5%) [7]. Unfortunately, the lifetime risk of BM in non-small cell lung cancer has been estimated at 40% [2], making clinical history one of the greatest prognosticators for BM. Had the patient not been undergoing regular surveillance magnetic resonance imaging of the head, his clinical diagnoses of BM may have been significantly delayed. Symptomatic presence of pituitary BM often presents as medial visual field cut secondary to cranial nerve impingement as seen in this patient.

Chemical indications of pituitary BM are severely limited. Pituitary stalk compression, a common side effect of pituitary metastases, may lead to elevated prolactin. Prolactinomas are well known to produce prolactin levels > 200 ng/mL, whereas pituitary stalk compression may present with a prolactin level between the upper limit of normal (13 ng/mL) and <200 ng/mL [8].  Table 1 demonstrates the decrease in prolactin production with relief of pituitary stalk compression after resection of the lung adenocarcinoma metastasis. Values between 20–200 ng/mL may be artificially low due to prolactin levels > 5000 ng/mL resulting in saturation and incorrect analyses of both capture and signal assay antibodies. This “hook effect” can be addressed with 1/100 dilution of the sample [9]. BM can additionally present with central diabetes insipidus with symptoms of both polyuria and hypernatremia, neither of which was seen in this patient.

The patient's bilateral medial visual field cut indicated acutely symptomatic BM. Initial treatment is both dexamethasone 4–8 mg/day and alleviation of the mass effect through surgical or stereotactic radiotherapy if feasible. The current literature does not recommend prophylactic anticonvulsant therapy [7]. The brain is a singularly difficult target for medical treatment as the blood-brain barrier (BBB) limits the efficacy of many types of chemotherapy, often creating a haven for metastases. However, there is ongoing debate in literature suggesting that the integrity of this BBB is compromised in the setting of these lesions. This patient's symptoms successfully resolved with surgery; however, further positron emission tomography revealed uptake in the area indicative of returning metastatic disease. Although treatments for BM are both variable and highly dependent on tumor type, it has been noted that donepezil has demonstrated to improve cognition, mood, and quality of life in these patients [7].

The patient demonstrated progression of disease on erlotinib, a 1st-generation EGFR tyrosine kinase inhibitor (TKI). Response rates of EGFR TKIs generally range from 70–80% with longer progression-free survival than previous standard chemotherapy regimens [7]. On average, the progression of disease occurs in 10–14 months due to new resistance mutations associated with EGFR-positive lung adenocarcinoma [10]. Initial screening for EGFR mutation from the lung core needle biopsies was performed with high-affinity class ribonucleotide analogs termed “locked nucleic acid probes” to identify wild-type EGFR and T790M mutations. However, this technique has a minimum sensitivity of 3% for T790M and 10–15% for L585R mutations [11]. There is emerging evidence to suggest that T790M mutation singularly develops during erlotinib treatment of EGFR-positive malignancies [12], and it is unlikely that the initial lung adenocarcinoma expressed T790M as the doubling time of the pituitary tumor was approximately 1 month and would have been visible far sooner on regular screening CT/MRI imaging. The patient's symptomatic pituitary mass was tested for T790M mutation through real-time PCR analysis [13] via the cobas EGFR Mutation Test P120019 (Roche Molecular Systems Inc., CA, USA) [14]. The patient's initial lung biopsy demonstrated the L858R EGFR mutation on exon 21 and when recurred was additionally identified with the T790M mutation on exon 20. This T790M mutation resulted in conformational change, sterically hindering erlotinib from binding to the adenosine triphosphate kinase pocket. Current EGFR TKIs include second-generation agents such as afatinib, dacomitinib, and neratinib or third-generation agents such as osimertinib, rociletinib, or olmutinib. Osimertinib in particular has been approved in T790M-positive non-small cell lung cancers (NSCLC), penetrates the BBB [15], and may offer this patient future benefit.

Unfortunately, neurosurgery could not be delayed due to mass effect of the underlying lung adenocarcinoma metastases on the pituitary and overlying optic chiasm. Had surgery been delayable, a far less invasive diagnostic test for T790M mutation could have been performed. The FDA has approved “liquid biopsy” [13] for EGFR mutations, which involves the testing of circulating cell-free tumor DNA (cfDNA) and has been demonstrated to reveal EGFR mutations not previously detected by biopsy in up to 34% of patients [16]. Moreover, this test can be performed on serum, rather than tumor tissue as previous ribonucleotide analogues have required. Given the rate of disease advancement on erlotinib [10], serial screens of cfDNA may play a future role in preempting changes in TKI treatment regimens before radiologic imaging reflects tumor progression. However, osimertinib has been associated with “pseudoprogression” or temporary paradoxical enlargement of EGFR T790M adenocarcinomas and may have worsened this patient's mass effect rather than relieved it [17].

In conclusion, physicians caring for patients with prior history of breast or lung cancer should remain vigilant in their history and physical exam for underlying signs of metastatic disease. AI is a rare complication of metastatic disease to the HPA axis and requires physiologic steroid replacement, ideally with hydrocortisone due to its lower LDL profile. Patients should be instructed to assess for signs of adrenal crises as soon as a diagnosis of AI is made as it can be life-threatening. Prolactin elevation < 200 ng/mL may be the first sign of pituitary stalk compression, and when an alarming clinical sign such as medial visual field cuts presents itself, patients should be started on dexamethasone dosing of 4–8 mg daily. The relief of mass effect is key in symptomatic pituitary BM and includes multidisciplinary involvement from specialties such as neurosurgery, as well as both medical oncology and radiation oncology. Given the recent advancements in tumor genomics and genetically targeted treatment modalities, BM pathology should be obtained via minimally invasive techniques provided the BM is not exerting mass effect on adjacent structures and requires surgical intervention. Finally, serum cfDNA may offer accurate nonsurgical screening for EGFR TKI resistance mutations and in turn facilitate changes in therapy to improve long-term patient outcomes.

## Figures and Tables

**Table 1:** Laboratory results before and after transsphenoidal pituitary metastases resection.

Hormone	Normal limit	Preresection	Postresection
Thyroxine	0.84–1.68 ng/dL	1.00 ng/dL	0.65 ng/dL
Thyroid-stimulating hormone	0.3–4.0 mIU/L	0.016 MCI/mL	0.019 MCI/mL
Follicle-stimulating hormone	1.6–17.8 mIU/mL	2.4 mIU/mL	0.8 mIU/mL
Luteinizing hormone	NR 1.8–12.0 mIU/L	0.05 mIU/mL	<0.1 mIU/mL
Testosterone	280–1100 ng/dL	<20 ng/dL	<20 ng/dL
Adrenocorticotropic hormone	9–52 pg/mL (2.0–11.0 pmol/L)	6.0 pg/mL	<5.0 pg/mL
Morning cortisol	>10 mcg/dL	<1.0 mcg/dL	<1.0 mcg/dL
Prolactin	<12.3 ng/mL (<0.55 nmol/L)	**28.9 ng/mL**	**14.9 ng/mL**
